# Supplementary material for: A systematic review of the 60 year literature: Effects of outreach programs in supporting historically marginalized and first-generation, low-income students in healthcare education
Source: PLoS One. 2022 Dec 1;17(12):e0278453. doi: 10.1371/journal.pone.0278453 (PMC9714932; doi:10.1371/journal.pone.0278453)
Supplement: S1 File — S1A Table: Barriers, assumptions, components, evaluative methods, conclusions and evidence-based success. S1B Table: Recommendations, reasoning, and conclusions of non-program high school healthcare education pipeline research. (DOCX) [file pone.0278453.s002.docx]

**SI: Study Characteristics, Bias, Results of Individual Studies, Certainty of Evidence**

**S1A: Barriers, assumptions, components, evaluative methods, conclusions and evidence-based success**

| **Article** | **Barriers** | **Theoretical Construct** | **Program Components** | **Duration (hours per year)** | **Evaluative Components** | **Evidence Based Success** | **Evaluative Improvement Needed** | **Bias** |
| --- | --- | --- | --- | --- | --- | --- | --- | --- |
| [**Patel (2015)**](https://www.mendeley.com/viewer/?fileId=2113cc0f-ef76-69eb-61e0-d3e20d8cf33c&documentId=fe6bc8a5-1102-396b-b76a-c2e9aecfd5d9) | SI, MSC, SE, AW, HCE | NO, - | ASP, UBP, HSC, MNT<1 | Not reported | SD, QM, PP, SI, CA | No | NO, SSLP, L>2, MC, QUS, SD, + | No |
| **Conclusion** | Increases were seen in mentee understanding of the premedical/medical school pipeline, intent to attend community college. Follow up study revealed all respondents had matriculated into college or community college  and still enrolled, 12 of 12 majoring in a healthcare education field, with all 12 college sophomores or juniors still intending to pursue medical school. The post-program surveys showed high levels of self-perceived  support, guidance, and positive experience from mentorship. | | | | | | | |
| [**Winkleby (2007)**](https://doi.org/10.1097/ACM.0b013e31802d8de6) | SI, CC, MSC, HCE, AW, AP | SD, + | URM, FGLI, SP, IE, UBP,  CA, HSC, PP, AW, MNT>1, SAT, E_B_, MC, SI, CC, I_F_, I_S,_ CL | 600 hours | L>10, QUI, QCA, QCG, QGA, QCE, QA | No | NO, MC, P/P, QI, QE | No |
| **Conclusion** | Suggest that the SMYSP summer residential program, distinguished by direct participation in the sciences, college admissions preparation, and long-term mentoring and career guidance, has been highly successful in reaching low-income students and preparing them for medical and other careers. Data from the program also highlight the need to track students for as long as 10–15 years to accurately assess college graduation rates and career choices. | | | | | | | |
| [**Crump (2015)**](https://link-springer-com.laneproxy.stanford.edu/content/pdf/10.1007/s10459-014-9540-6.pdf) | EXP, SE, MSC, CC | SD, + | SP, IE, CA, E_B_, MNT>1, MC, CC, SI | 600 hours | QCG, QSTEM, QCA, QGA | No | C, MC, P/P, SSLP | No |
| **Conclusion** | Due to inadequate matching of the control group and low power resulting from small sample size, meaningful differences were not observed. Lessons learned for future studies. | | | | | | | |
| [**Derck (2016)**](http://sfx.stanford.edu/local?sid=stanford:laneweb-search-pubmed&id=pmid:28406112) | MSC, SI, AP | MND, - | URM, SP, ASP, UBP, MC, E_B_, I_S,_ M_S,_ MNT<1, SI, IQB | 48 hours | QUF, QUI, QUS | No | SD, +, L>4, P/P, QCA, QCG, QA, QE, QI, QMS | No |
| **Conclusion** | Key qualitative themes pertained to personal identity and its perceived effect on goal achievement and  positive affect of direct mentorship and engagement with current healthcare providers. | | | | | | | |
| [**Baker (2014)**](https://pdfs.semanticscholar.org/d636/6a5686ae98ea5931ce1e6c732cee680fd820.pdf?_ga=2.227034013.1203804911.1597220380-1450757183.1597220380) | $$, AP, MSC | SD,  + | $$, SP, URM, FGLI, E_M_, T, CL, FT, SI, MNT>1, CC, SS, PP | UNK | IC, L, SD, QCG, QM, QE, QUS, QWO | **No** |  | No |
| **Conclusion** |  | | | | | | | |
| [**Holden (2015)**](https://web-b-ebscohost-com.laneproxy.stanford.edu/ehost/detail/detail?vid=0&sid=8c3497e6-ea4d-47c7-b888-8ecc1afc7c14%40pdc-v-sessmgr04&bdata=JnNpdGU9ZWhvc3QtbGl2ZSZzY29wZT1zaXRl#AN=109101232&db=bah) | IM, PA, SI, SE, HCE | SD, + | AS, HSB, I_F_, I_S_, E_B_, MC, PP, FT | 18 hours | SD, QE, QK, QM, QA, QUS | No | NO, L, P/P, MC, QCA, QCG, QSTEM | No |
| **Conclusion** | Evaluation data indicate this program helped the students develop the characteristics necessary for a successful health career, including knowledge acquisition, self-efficacy and positive attitudes toward a career in health. | | | | | | | |
| [**Phillips (1981)**](https://journals.lww.com/academicmedicine/Abstract/1981/09000/Minority_recruitment_to_the_health_professions__a.6.aspx) | AW, IM, HCE | SD, + | URM, FGLI, CA, SP, UBP, E_B_, MC, FT, SI, AW, CC, SS, CL | UNK (15 days) | L>4, MC, SD, QE, QCE | No | SSLP, LRR | No |
| **Conclusion** | After 6 years of follow-up, the authors found that program participants were four times more likely than non-participants to be employed  in a health career independent of the effect of obstacles on their careers. In regards to those with the PG and CG who were employed in healthcare careers, those among both groups who had been educated in underserved areas were more likely to remain in that area to practice. However, a larger percentage of the PG students went to AHEC area schools for healthcare education and thus yielded a much larger percentage of their group who ultimately practiced healthcare in their underserved area. | | | | | | | |
| [**Perry (1976)**](http://ovidsp.ovid.com/ovidweb.cgi?T=JS&PAGE=reference&D=ovfta&NEWS=N&AN=00004999-197603000-00003.) | AW, IM, SS, HCE | SD, + | URM, FGLI, SP, UBP, E_B_, MC, FT, SI, AW, CC, SS, CL | UNK (15 days) | SD, MC, QCE, QCA | No | L>2, P/P, SSLP, LRR, NSS | No |
| **Conclusion** | Differences in college enrollment were not significant; however, there was a significant difference on stability of first career choice for PG . The study makes it clear that program effects cannot be assumed unless a follow-up evaluation is undertaken. | | | | | | | |
| [**Fernandez (2018)**](https://link.springer.com/article/10.1186/s12909-018-1205-3?utm_source=getftr) | EXP, HCE, MSC | MND, + | URM, UBP, SP, FT, E_B_, MNT<1, M_F_, MC, SI | 49 hours | P/P, QK, QM, SSLP, LRR, NSS | No | NO, MC, SSLP, LRR, NSS | No |
| **Conclusion** | Due to clear experimental design flaws such as no control group, inadequate response rate, inadequate sample size resulting in inadequate power, and lack of statistical significance for many measures this studies conclusions do not add to evidence-base for practice. | | | | | | | |
| [**Thurmond (1990)**](https://journals.lww.com/academicmedicine/Abstract/1990/07000/Minority_students__career_choices_and_education.17.aspx) | AP, IM, SS, CC, MSC | NO, - | URM, CA, SP, IE, UBP, CC, MY,E_P_, E_B_, E_M_, I_F_, I_S,_ MC, MNT<1, M_S_ | 8 weeks + 3 days | L>4, AD, SD, QA, QCA, QCG, QGA, QCE | No | NO, MC, P/P | No |
| **Conclusion** | Although the lack of a comparison group prevents meaningful attribution of success to intervention over factors such as selection bias or other available interventions, this study still revealed that 100 of 101 (99%) had enrolled in college, 71 of 101 graduated college (70%) and 16 were still in college, 39 had chosen science majors. Of those, 24 students were medical doctors in residency or medical students and 10 students were working in other health related fields (E.G. nursing). Total of 34 students in healthcare fields and 16 still in school. 37 college graduates had gone into non-healthcare fields after graduation. | | | | | | | |
| [**Thurmond (1999)**](https://journals.lww.com/academicmedicine/Abstract/1999/04000/Why_students_drop_out_of_the_pipeline_to_health.44.aspx) | AP, IM, SS, CC, MSC | NO, - | URM, CA, SP, IE, UBP, CC, MY, I_F,_ I_S_, E_P_, E_B_, E_M_, MC, MNT<1, M_S_ | 8 weeks + 3 days | L>4, AD, SD, QUS, QA, QCA, QCG, QGA, QCE | No | NO | No |
| **Conclusion** | The most frequent reasons cited by students in this survey were (in the order of frequency): loss of interest, fear of problems with grades, feeling of inadequate preparation in science (especially chemistry), other fields/majors internships, summer jobs, and mentorships stimulated other interest areas, recruitment by other fields/majors (especially engineering), scholarships in other fields/majors, long time required to achieve health career, distaste for illness, new interests and career possibilities arising from other courses. Summer programs are successful, but may not be enough to help students with difficult science courses in college, especially chemistry. Another important conclusion is that much more needs to be done to financially incentivize persistence in the healthcare pipeline and to help students find mentors with whom they can develop relationships and to give them opportunities to work in health care settings and develop social capital necessary for LOR’s and opportunities. | | | | | | | |
| [**McKendall (2000)**](https://journals.lww.com/academicmedicine/Fulltext/2000/10001/The_Health_Sciences_and_Technology_Academy_.39.aspx) | $$, SE, EXP, AP | SD, + | URM, FGLI, SP, UBP/HBP, ASP, E_P_, I_F_, $$, IQB, CL | (UNK) 4 years | SD, QGPA, QM, QE, QA, QUS | No | L>2, P/P, IC, MC, QCA, QCG | No |
| **Conclusion** | Although this program had a strongly theoretical foundation , persuasive qualitative content, and robust longitudinal program with revolutionary scholarship opportunities and inquiry based academic preparation by paid educators with integration for high school science teachers, it lacked an appropriate comparison group because they matched based on “same status” which was defined as the same year in college, same major, and state resident status. A matched comparison group would have also included matching for application to the program (not accepted), URM status, household income, and parents education at a minimum. In conclusion, this program appears to be successful but lacks the valid proof of evidence based success which would allow other states to be able to defend the significant  investment of money and resources to replicate it. | | | | | | | |
| [**Fincher (2002)**](https://journals.lww.com/academicmedicine/Fulltext/2002/07000/Health_Science_Learning_Academy__A_Successful.23.aspx) | AP | NO, - | URM, CA, UBP, ASP, E_B_, E_M_, SAT | 54 hours | AD, QSAT, P/P | No | IC, L>4, MC, QCA, QSTEM, QCG, | No |
| **Conclusion** | The mean SAT score for students who completed the HSLA program was 1,066, compared with a mean of 923 for all college-bound students in the participating schools. The mean increases in SAT scores for students who completed the four-year program were .5% (1,100 to 1,105) for students attending a magnet high school and 18% (929 to 1,130) for students attending the comprehensive high school. The mean overall increases in SAT scores for students in the two high schools were 1% (1,044 to 1,048) and 9.1% (765 to 834), respectively. | | | | | | | |
| [**Butler (1991)**](https://journals.lww.com/academicmedicine/Abstract/1991/06000/Baylor_s_program_to_attract_minority_students_and.1.aspx) | AP, HCE, IM | NO, - | URM, HBP, E_P_, E_B_, E_M_, I_F_, MC, SI | UNK | L>10, SD, HGRAD, CADMIT, CGRAD, CSTEM, GA, CE | No | NO, P/P, MC, LRR | No |
| **Conclusion** | This article summarizes six high school pipeline programs developed in conjunction with Baylor University  but the only program with study data had no comparison group aside from comparison with national averages. This is a major design flaw that prohibits attribution of the outcomes seen to the program due, in part, to selection bias for the program and self selection bias by the type of student that would be motivated, supported, or have ample connection to resources to take advantage of applying to such a program. Use of a MC group of students who had applied but not been accepted would have been a close comparison while randomization of selected students and control students possessing requisite first-gen, low income, or underrepresented status would have been even more useful and appropriate as long as there were two to three times more applicants fitting this criteria than spots available. | | | | | | | |
| [**Beck (1991)**](https://journals.lww.com/academicmedicine/Abstract/1978/08000/Recruitment_and_retention_program_for_minority_and.4.aspx) | IM, HCE, SE, EXP, AW | No, - | URM, FGLI, ASP, UBP, CA, MC, E_B_ | 10 credit hours | QHG, QCA | No | NO, MC, P/P | No |
| **Conclusion** | Has a section regarding University of Colorado School of Medicine’s high school program from 1969-1977. Sparse data accompanies it with no comparison group and high probability for selection bias that ruins attribution of outcome to intervention. | | | | | | | |
| [**Jones (1978)**](https://journals.lww.com/academicmedicine/Abstract/1990/11000/New_York_s_statewide_approach_to_increase_the.2.aspx) | AP, IM, AW, CC, PA, HCE | SD, + | URM, SP/ASP, UBP, I_F_, PP, MS, CC, FT, MC, SI, SS, SAT | UNK (Saturday sessions) | SD, QCA, QCS | No | NO, LRR, P/P, MC, L>4, QUS | No |
| **Conclusion** | The Science and Technology Entry Program (STEP) is a high school pipeline program which 12 New York public and private medical schools participate in with their own various program designs centered around broad guidelines.  The study discussed in this article lacks a comparison group, pre/post measures, appropriate response rate, qualitative data, or adequate longitudinal follow-up. | | | | | | | |
| [**Rosenbaum (1993)**](https://onlinelibrary.wiley.com/doi/abs/10.1096/fj.06-7845lsf) | MSC, AP, SE, SI, EXP | SD, + | IQB, MNT<1, FM, EXP, SI, SP | 36 hours | SD, MC, QM, QCS | No | P/P, LPSS | No |
| **Conclusion** | This was not a URM pipeline program, it was a STEM pipeline program open to all, but had this study been designed with a pre/post survey methodology, the data would be both enlightening to the field and attributable to their intervention. Unfortunately, the authors did not include a pre program survey and have a questionable match for their comparison group. | | | | | | | |
| [**Davis (1982)**](https://journals.lww.com/academicmedicine/Abstract/1982/07000/The_Med_COR_study__preparing_high_school_students.4.aspx) | AP, IM, CC, HCE, MSC, PA, SI, SE, EXP | SD, + | URM,  UBP, CA, SP/ASP, UBP/HBP, CL, PP, MC, CC, SAT | UNK | NO, SD, QGPA, QCA, QCG, QGA, QCS | No | MC, P/P, LRR | No |
| **Conclusion** | Although this study lacked the necessary design to attribute the outcome to the intervention, all 240 respondents (50% response rate) were admitted to college and 68% of  the programs college graduates were in a graduate health education program. | | | | | | | |
| [**Slater (1991)**](https://journals.lww.com/academicmedicine/Abstract/1991/04000/A_program_to_prepare_minority_students_for_careers.11.aspx) | AP, MSC, SS, SI, SE | SD, + | URM, HBP, IDS, CA, I_F_, E_B_, E_M,_ MNT>4, T, FT, SI, SS, PP | UNK (4 year HBP) | IC, P/P, AD, QSAT, QCA, QHG, QSTEM | No | MC, QMS, QCS, QCG, QGA | No |
| **Conclusion** | Although this study lacked the necessary design to attribute the outcome to the intervention due to inappropriate comparison groups to negate the selection bias inherent in this program, the authors noted that graduates of this selective program graduated high school and were admitted to college at higher rates than peers at their school, in the state, and nationally. | | | | | | | |
| [**Katz (2016)**](https://www.ncbi.nlm.nih.gov/pmc/articles/PMC4724384/) | MSC, AP, EXP, AW | SD, + | URM, SP, IE, CA, E_B_, E_M_, EXP | 240 hours | P/P, QK, QM | No | MC | No |
| **Conclusion** | Psychometric analysis indicated poor model fit for a 1-factor model for the total scale and majority of subscales. Non-parametric tests indicated statistically significant increases in 13 items and decreases in 2 items. Students did not have an accurate view of nursing and college, and underestimated support needed to attend college. However students realized that nursing was a profession with autonomy, respect, and honor. | | | | | | | |
| [**Habig (2018)**](https://link.springer.com/article/10.1007%2Fs11165-018-9722-y) | AP, EXP, MSC | SD, + | URM, SP/ASP, IQB, MNT>4 | UNK (4 year MBP) | L>4, QMS, QCE, QUS, QUI, QUF | Yes, QUAL  No, Q | IC, MC, P/P | No |
| **Conclusion** | Through a thorough theoretical underpinning and rigorous qualitative analysis the authors were able to provide compelling evidence of the mechanism by which the Lange intervention increased interest, academic success, and persistence in STEM education. However, the quantitative data lack validity for attribution of outcomes to intervention due to the lack of an appropriate comparison group as defined above. | | | | | | | |
| [**Wallace (2015)**](https://link.springer.com/article/10.1007/s10956-014-9536-2) | EXP, SI, HCE, SS, MSC, PA, AP | MND, - | URM, FGLI, CA, SP, CL, FT, MNT<1, EXP MC, M_s_, SP, SS, PP, E_B_, E_C_, E_M_ | 60 hours | P/P (K only), K, SD, QUAL | No, QUAL | MC, P/P, L>4, | No |
| **Conclusion** | While the authors found that greater participation in TMA significantly predicted greater interest in medical and allied health careers their study was not designed to test program impact, nor did it examine academic outcomes. The only P/P measure related to program specific knowledge lacks sufficient evidence of long term contribution to pipeline persistence. The qualitative data pertained to whether participants liked the sessions. The qualitative study focused on program improvement and did not  examine intervention attributions to outcome. One qualitative question was an exception, and succeeded in examining attributions to outcome by exploring  how each session  influenced their attitude about future careers in health care. | | | | | | | |
| [**Gefter (2018)**](https://doi.org/10.1007/s40615-017-0414-5) | HCE, IM, MSC>1 | MND, - | AS, GS, FT, MST, MNT<1, E_B_, SP | 22.5 | SD, QA, QK | No | NO, P/P, L, MC, QCA, QCG, QI, SD, AD, QUF, QUI | No |
| **Conclusion** |  | | | | | | | |
| [**Bidwell (2019)**](https://jamanetwork.com/journals/jamanetworkopen/article-abstract/2748656) | HCE, IM, SE, AW | NO, - | SP, FT, MST, I_S_, I_F_ | 7.5 | P/P, SD, QA, QUS | Yes | L, QSTEM, | No |
| **Conclusion** |  | | | | | | | |
| **Barriers Key:**  $$= Financial support  AP= Academic preparation  PA= Parental awareness  IM= Interest and motivation  SI= Scientific identity  SE= Scientific efficacy  MSC= Mentorship and social capital  AW= Awareness of requirements  HCE= Health career exposure  EXP= Science experience  SS= Academic study skills  CC= College Prep Counseling  **Theoretical Underpinning Key :**  NO= No theoretical construct discussed  MND= Mentioned but not explicitly detailed  SD= Specifically detailed theoretical construct  += Outcome tied to theoretical construct  - = Outcome not tied to theoretical construct | | **Components Key :**  FGLI=Targeted for first gen college graduate or low income  URM= Targeted for underrepresented minority preferential  SP=Summer program  IE= College immersion experience > 1 day  DS=During school program  AS= After school program  MY= Multi year opportunity  HSC= High school credit  CA= Competitive application  IQB= Inquiry based  MNT<1= Mentoring less than one year  MNT>1= Mentoring greater than one  year  M_F_= Faculty mentor  M_S_= Student mentor  I_F_= Faculty instruction  I_S_= Student volunteer instruction  T= Tutoring  CL= Collaborative learning  MS= Mindset training  SS= Study skills training  MC= Medical content exposure  E_C_=Chemistry enrichment  E_C_= General enrichment  E_G_= Physics enrichment  E_B_= Biology enrichment  E_M_= Math enrichment  SAT= SAT/ACT prep  EXP= Science lab experience  SP=Scholarly presentation  PP= Parental programming  AW= Alumni workshops  CC= College admission/application counseling  CF= College financial counseling  FT= Field trips  SI= Shadowing or internship experiences  $$= Financial scholarship | | | **Evaluation Methods Key:**  CG= Comparison group  P/P= Pre and post data  L>2= Longitudinal>2 years post program  L>4= Longitudinal>4 years post program  L>10= Longitudinal>10 years post program  SD= Survey data  AD= Administrative data  QUAL= Qualitative interview or focus group | **Evaluation Measures Key:**  GPA= GPA  HGRAD= high school graduation  SAT= SAT or other standardized test data  CADMIT= college admission  CG= college graduation  CS= college STEM major  GA= graduate school admission  CE= health career employment  SE= Student evaluation  K= knowledge of subject matter  CSTEM= STEM courses taken in high school  WO= work obligation  MOT= motivation/interest  ATT= attrition  ID= identity  EFF= efficacy | **Evidence Based Success Key:**  **Evaluative Shortcomings:**  M_NO_= No comparison group  M_IC_= Inappropriate comparison group  M_LRR_= Low response rate  M_SSLP_= Small sample low power  M_NSS_= Not statistically significant  M_P/P_= Pre and post data needed  M_QUAL-S_= Qualitative survey needed  M_QUAL-I_= Qualitative interview needed  D_STEM_= STEM courses or extracurriculars taken in high school  D_CADMIT_= Recommend college admission  D_CGRAD_= college graduation  D_STEMMAJ_= college STEM major  D_GRADADMIT_= graduate school admission  D_HCEMPLOY_= health career employment  D_MENTSUPP_= mentor support  D_WORK_= work obligation  D_MOTIV_= motivation/interest  D_ATTRIT_= attrition data needed  D_IDENT_= identity data needed  D_EFF_= efficacy | |

**S.1B: Recommendations, reasoning, and conclusions of non-program high school healthcare education pipeline research**

| **Article** | **Shortcoming Addressed** | **Theoretical Framework** | **Research Methodology** | **Evaluative Improvement Suggested** | **Bias** |
| --- | --- | --- | --- | --- | --- |
| [**Freeman (2016)**](https://doi.org/10.1097/ACM.0000000000001020) | Qualitative research detailing barriers to URM persistence in healthcare education pipeline. | **N/A** | QUF |  | No |
| **Conclusion** | Students described challenges including inadequate institutional resources (e.g., sparse clinical opportunities), strained personal resources (e.g., conflict arising from familial pressure), inadequate guidance and mentoring to assist with key career decisions, and societal barriers. For participants, these challenges caused them to question the viability of persisting in the pipeline to a medical or dental career. | | | | |
| [**Winkleby (2014)**](https://www.ncbi.nlm.nih.gov/pmc/articles/PMC3927943/) | Design and methods of a matched control group pipeline study | SD, + | QCG, QSTEM, QCA, QGA | MC, P/P, SSLP | No |
| **Conclusion** | Minimal loss to follow-up. High potential for effective study design, but flaws in the matching system. | | | | |
| [**Carline (1998)**](https://doi.org/10.1097/00001888-199803000-00018) | Not a program | N/A | LR | QCA, QCG, QSTEM, QE, QI, QA, QMS | No |
| **Conclusion** | Reviewed 19 articles describing 27 programs published from 1966 to 1996 to identify enrichment programs for URM precollege students. Recommended improvements to program evaluations. Evaluators should adopt common terminology for activities and outcomes. Participants' economic and educational disadvantages should be described. Programs' theoretical underpinnings should be identified and related to evaluation. Measures should include immediate effects as well as long-term outcomes. Where possible, data from comparison groups should be reported to support conclusions. Adequate funding needs to be available to design and complete reasonable evaluations. | | | | |
| [**Carline (2003)**](https://doi.org/10.1097/00001888-200305000-00008) | Best practices for partnerships between public schools and medical institutions | N/A | QUS, SD |  | No |
| **Conclusion** |  | | | | |
| [**Cooper (2003)**](https://journals.lww.com/academicmedicine/Fulltext/2003/09000/Impact_of_Trends_in_Primary,_Secondary,_and.4.aspx) | Impact of Trends in Primary, Secondary, and Postsecondary Education on Applications to Medical School | N/A | SA, QA, QCA, QCG, QGA, QCE |  | No |
| **Conclusion** | Recommended: More financial aid, affirmative action, and more rigorous K-12 public education. | | | | |
| [**Hosbein (2020)**](https://pubs.rsc.org/en/content/articlehtml/2020/rp/c9rp00193j#:~:text=Alignment%20of%20theoretically%20grounded%20constructs%20for%20the%20measurement%20of%20science%20and%20chemistry%20identity,-Kathryn%20N.&text=Identity%20has%20been%20theorized%20to,chemistry%20person%20within%20the%20classroom.) | Development of an assessment of chemistry identity | SD,  + | QUI | QI, QA, QE | No |
| **Conclusion** | Scientific identity was defined, through qualitative analysis using existing theoretical constructs, as consisting ofÑ mindset,situational interest, verbal persuasion, vicarious experiences, and mastery experiences. | | | | |
| [**Graham (2019)**](https://doi.org/10.3102/0002831219842571) | MSC | SD, + | QG, QM, QMS, QUS | L>2 | No |
| **Conclusion** | Results revealed a positive relationship between mentorship, mentorship experiences and college adjustment, and an inverse relationship with impostorism. Furthermore, students with mentors reported significantly higher belongingness and college adjustment scores compared to students with no mentors. | | | | |
| **Salehi (2020)** | AP, SI | SD, + | SD, QK, QSTEM, QM, QI, QPS | L>2, QCG, QGA | No |
| **Conclusion** | Lower incoming preparation largely explained why URM and Fgen students underperformed on both exams and total grades in introductory courses across both colleges. Recommended: Enhanced AP for FGLI and URM students whether by supplementary instruction or developmental preparatory coursework in gateway STEM courses. | | | | |
| [**Trujillo (2014)**](https://www.lifescied.org/doi/10.1187/cbe.13-12-0241) | SI, SE | SD, + | LR |  | No |
| **Conclusion** | Explored three affective constructs that may be important for understanding biology student learning: self-efficacy—the set of beliefs that one is capable of performing a task; sense of belonging—when one feels a part of a particular group; and science identity—the extent to which a person is recognized or recognizes himself or herself as a “science person.” | | | | |
| [**Camacho (2017)**](https://journals-sagepub-com.laneproxy.stanford.edu/doi/full/10.1177/0163278715617809) | $$ | SD, + | SA | L, MC, P/P | No |
| **Conclusion** | Results from this evaluation showed that a total of 78,316 scholarships totaling over US$213 million were awarded to students from disadvantaged backgrounds between AY 2008–2009 and AY 2012–2013. Over this 5-year period, scholarships were awarded to students in over 30 different types of health professions training programs, and more than half of all scholarships awarded went to students who are considered URM in their health professions. By the end of AY 2012–2013, over 18,000 students who received a scholarship through the SDS program graduated from their health professions training programs. While these statistics show that the SDS investment supports efforts to increase the supply of new health professionals, additional prospective studies will be needed to accurately gauge the contribution of the SDS program on the recruitment and retention of students from disadvantaged backgrounds. For example, at this time, we cannot ascertain that students who received a scholarship would have otherwise not attended their health professions training program and, therefore, cannot attribute their enrollment and graduation to the SDS program. | | | | |
| [**Felix (2004)**](https://www.mendeley.com/catalogue/5056d837-58e5-35fc-b94d-13121c5abe03/?utm_source=desktop) | Does HHMI funding help science outreach programs? | No, - | MC (nearly-accepted programs), QA | P/P | No |
| **Conclusion** | HHMI Funded programs produced more products and more products per user. | | | | |
| **Barriers Key:**  $$= Financial support  AP= Academic preparation  PA= Parental awareness  IM= Interest and motivation  SI= Scientific identity  SE= Scientific efficacy  MSC= Mentorship and social capital  AW= Awareness of requirements  HCE= Health career exposure  EXP= Science experience  SS= Academic study skills  CC= College Prep Counseling  **Theoretical Underpinning Key :**  NO= No theoretical construct discussed  MND= Mentioned but not explicitly detailed  SD= Specifically detailed theoretical construct  += Outcome tied to theoretical construct  - = Outcome not tied to theoretical construct | | | **Evaluative Shortcomings:**  HA= High attrition  LRR= Low response rate  SSLP= Small sample low power  NSS= Not statistically significant  P/P= Pre and post data needed  QSTEM= Quantitative STEM courses or extracurriculars taken in high school  QCA= Quantitative college admission  QCG= Quantitative college graduation  QCS= Quantitative college STEM major  QGA= Quantitative graduate school admission  QCE= Quantitative health career employment  QMS= Quantitative mentor support  QWO= Quantitative work obligation  QM= Quantitative motivation/interest  QA= Quantitative attrition  QI= Quantitative identity  QE= Quantitative efficacy  QUS= Qualitative survey  QUI= Qualitative interview | | |
