# Supplementary material for: A systematic review of the 60 year literature: Effects of outreach programs in supporting historically marginalized and first-generation, low-income students in healthcare education
Source: PLoS One. 2022 Dec 1;17(12):e0278453. doi: 10.1371/journal.pone.0278453 (PMC9714932; doi:10.1371/journal.pone.0278453)
Supplement: Attachment — Submitted filename: Response Letter(1).docx [file pone.0278453.s003.docx]

Dear editor and reviewers,

We greatly appreciate the careful consideration taken to improve the presentation and content of the manuscript. We believe the editor and reviewers’ comments were essential to strengthen the overall presentation and scholarship of the work. We have provided a table below summarizing the feedback from the editor and reviewers as well as responses and action from our team.

**Feedback from the editor and our response:**

We thank you for suggesting these edits and have implemented all changes accordingly.

| Feedback | Response and Action |
| --- | --- |
| Please complete your Competing Interests on the online submission form to state any Competing Interests. If you have no competing interests, please state "The authors have declared that no competing interests exist.", as detailed online in our guide for authors. | The change has been made in the main document (Page 19):  “The authors have declared that no competing interests exist.” |
| Please ensure that you refer to Figure 1-3 in your text as, if accepted, production will need this reference to link the reader to the figure. | The change has been made in the main document (Pages 4-5):  “Due to the small initial turn-out of articles related to the keywords, the search was expanded to include *all* articles regardless of publication date. Our second search returned 55 relevant articles from the journals of Academic Medicine, Medical Education, Medical Teacher, and BMC Medical Education of which 18 were excluded due to a lack of reported findings with qualitative, quantitative, or mixed qualitative and qualitative data (see Fig 1).”  “The majority of the research studies (N=19) are from the past ten years (see Fig 2) and the majority of research studies (N=12) from the Journal of Academic Medicine (see Fig 3).” |
| Please include captions for your Supporting Information files at the end of your manuscript, and update any in-text citations to match accordingly. Please see our Supporting Information guidelines for more information: | The change has been made in the main document and in the Supporting Information (Page 20):  Supporting Information S1A:Barriers,assumptions, components, evaluative methods, conclusions and evidence-based success  S1B: Recommendations, reasoning, and conclusions of non-program high school healthcare education pipeline research |

**Feedback from reviewer 1 and our response:**

We thank you for the feedback and the time to also add page and line numbers to the review, which has allowed us to implement the changes easily. We also agree the writing of the paper needed to be greatly improved, so we revised the entirety of the manuscript. We have implemented all changes accordingly.

| Feedback | Response and Action |
| --- | --- |
| My primary concern is with the written English of the manuscript. There are many places throughout the manuscript where the sentence structure, subject-verb agreement and use of prepositions confuse the meaning of the sentences. Overall, this has a substantial impact on the points the authors wish to communicate. | We agree and sincerely apologize for the written English of the manuscript upon submission. We have significantly re-written every section for clarity. We have also consistently used the same verbiage (e.g., studies on healthcare education outreach programs) when explaining the same phenomenon in the text. |
| The authors do not adequately draw on the literature to establish the link between underrepresentation in healthcare and gaps in quality care outcomes for marginalized groups. I think more needs to be done to explain this. | We agree and the change has been made in the main document (Page 2):  “However, healthcare professionals who are first-generation Americans, persons from low socioeconomic backgrounds, and Black, Indigenous, People of Color (BIPOC) have greatly contributed to how medical conditions such as strokes, heart attacks, and cancer impact subpopulations differently, which reflects how compassion and cultural competency often originate at the intersection of lived experience and medical training(4). Thus, researchers have hypothesized healthcare disparity can be addressed by supporting students who are first-generation Americans, persons from low socioeconomic backgrounds, and underrepresented racial and ethnic minorities (hereafter, historically marginalized students) to become healthcare professionals(4) since historically marginalized students are often multilingual(5), have financial knowledge from navigating the healthcare system from the position of a person from a low socioeconomic background(6) and have the necessary compassion and cultural competency(7). Unfortunately, a massive *education disparity* also exists for historically marginalized students as compared to their historically non-marginalized counterparts to pursue healthcare professions(8).” |
| The UMMC study should be described in greater detail because the primary purpose of the manuscript is to critically evaluate the existing literature. The reader needs to be convinced that the results of this study are believable given all of the shortcomings that are identified in the body of the manuscript. | We agree and the change has been made in the main document (Page 2):  “The study at UMMC drew from the Mississippi Science Framework that theorized program components such as student research mentorship, teacher professional development, disciplinary content knowledge enrichment, and practical experiences, which has promoted sustainability of the program for 28 years(22).” |
| Study Risk of Bias Assessment: the explanation of how this was done is not at all clear to me. I cannot distinguish between this explanation and the explanation for data items, quality, and eligibility assessment. | We agree and the change has been made in the main document (Page 4). We also cite (Page 16) how the review of bias by seeing if studies had reported any conflicts of interest is a limitation of the work because it is not an encompassing approach:  “After the abstracts were reviewed, the first author coded the studies for inclusion and all three authors worked collaboratively to verify the inclusion of each study for review. We examined the articles for their quality of 1) design, 2) evaluation, and 3) analysis and documented features of each article in tables (see supplemental information). To examine bias within the articles reviewed, we documented if authors declared any conflicts of interest.” |
| I think more needs to be said about the fact that "lack of study results" was a criterion for excluding articles from analysis. Do you mean quantitative results? I think it is an important oversight that you potentially missed qualitative results in a number of the articles that the search returned. There are only 120 total articles, so I think some review should be done to ensure that you did not miss qualitative results in the excluded articles. | We agree and for clarity, the studies needed to be classified as research studies by the journal (e.g., not editorials or tutorial papers) with defined methods, data, and findings sections. Studies needed to define the data collected and how the data was analyzed rather than a summary of what the components of the healthcare education outreach program did, which was the case for the papers excluded. We also cite this is a limitation of the work (Page 16). The change has been made in the main document (Page 4-5):  “Our second search returned 55 relevant articles from the journals of Academic Medicine, Medical Education, Medical Teacher, and BMC Medical Education of which 18 were excluded due not being classified by the journal as a research article containing reported findings with qualitative, quantitative, or mixed qualitative and qualitative data (see Fig 1). To be eligible for review, the articles needed to be research studies, which have explicit methods, data, and findings sections.” |
| Results section, first sentence. I think you need to justify this claim with data or literature. | We agree and the change has been made by citing the literature moving away from the pipeline metaphor (page 9):  Moving away from the pipeline metaphor and towards sustainable progress, studies on healthcare education outreach programs should 1) understand the main barriers to historically marginalized students entering, persisting, and succeeding in healthcare professions; 2) the protective factors that are correlated with increased entry, success, and persistence into healthcare professions; and finally, 3) the components that can successfully mitigate barriers or facilitate protective factors(18-22). |
| (Minor) Some figures are reported to three decimal places and others to two. Please be consistent. | We agree and have changed the text and figures to include two decimal places. |
| You never explain the concept of social capital, but draw on it several times in the manuscript to justify claims. Please provide some context. | We agree and the change has been made in the main document (Page 17):  “Mentorship often provides historically marginalized students with social capital, which is a sociological term from Pierre Bourdieu that shows how access to knowledges, resources, and experiences can better facilitate social navigation(57).” |
| "Trends in the performance outcomes" -- if these are gaps in outcomes, please say so. It is not clear form this sentence that the outcomes are undesirable. | We agree and have removed the phrasing from the main document. |
| what does it mean for qualitative results to "challenges the power" required for quantitative analyses? | We agree and have removed the phrasing from the main document. |

**Feedback from reviewer 2 and our response:**

We thank you for the feedback, especially phrasing the feedback in questions we can answer in our manuscript and response. We also appreciate the direct feedback on methodological considerations that are vital to maintaining research integrity and validity. We have implemented most changes accordingly.

| Feedback | Response and Action |
| --- | --- |
| Although there is variation in systematic reviews regarding their inclusion, a guiding research question (or questions) could be helpful to more clearly articulate the focus of the review. Based on the analysis and framing, it is clear the authors have a specific goal with reviewing the literature (i.e., evaluating overall quality of research design), which could be communicated through research question(s). | We agree and the change has been made in the main document (Page 3):  Our systematic literature review seeks to answer:   - How are studies on healthcare education outreach programs designed by drawing from theory? - How are studies on healthcare education outreach programs evaluated using quantitative, qualitative, and mixed-methods? - How are studies on healthcare education outreach programs analyzed to generate reliable and generalizable results? |
| One area for improvement would be the ways in which the data analysis is discussed. Data analysis is briefly discussed in the paper, with a supplemental document providing more insight into the nature of the analysis (this supplemental paper was very helpful). After reading the paper and the supplemental, it is not clear what the coding process looked like. Would you consider each acronym in the supplemental to be a code (e.g., NO, ASP, UBP, HSC, etc.) or were the codes larger in grain size (bullet points under the criteria in Figure 4)? There are also other themes that might suggest additional codes (e.g., Figure 5 – “specifically detailed”, “not specifically detailed” – but this wording is different from the wording in the supplemental under “Theoretical Underpinning Key”, so it is unclear if they are distinct or if they map onto one another; other examples would be the other descriptive trends in the other figures like Figure 9, etc.). | We agree and for clarity, we do consider each acronym to be a sub-code and they map onto the larger scheme for design, evaluation, and analysis. For the main text, we will only include the larger code category and how it aligns with the design, evaluation, and analysis to streamline the paper. We also changed the figure heading to match better what is in the Supplemental and the paper. The changes have been made in Figure 4 where we have the research question, coding features, and adequate quality criteria. |
| Also, was it one author coding or multiple authors coding and did they calculate any measures related to reliability for the code assignments? | We agree and for clarity, the first author listed coded through the literature and the second and third author listed verified the codes with the first author. Disagreements were discussed together until reaching consensus. The change has been made in the main document (Page 3-5) in Table 1 and Study Search, Retrieval, Eligibility, and Selection. |
| Due to the small sample size, I would suggest moving away from quantifying the findings, particularly, reconsider reporting percentages. The percentages can be misleading because they distance the findings from the context. This is particularly the case when there is discussion about percentages involving a subset of the sample (e.g., “Of the 54.2% of studies with detailed theoretical frameworks, 54.2% had study goals that aligned with the framework”). For transparency and clarity, provide raw values instead of percentages (“n=20 of the studies had a detailed framework, with only n=11 of these studies having goals that aligned with the framework”). This suggestion applies across the findings, including reporting values in figures, tables, etc. | We agree and have also decided to include the number of articles in each of the titles so that it is transparent for the reader to calculate. |
